# Basic life support and automated external defibrillator skills among ambulance personnel: a manikin study performed in a rural low-volume ambulance setting

**DOI:** 10.1186/1757-7241-20-34

**Published:** 2012-05-08

**Authors:** Anne Møller Nielsen, Dan Lou Isbye, Freddy Knudsen Lippert, Lars Simon Rasmussen

**Affiliations:** 1 Department of Anaesthesia, Centre of Head and Orthopaedics, Copenhagen University Hospital, Rigshospitalet, Blegdamsvej 9, Copenhagen 2100, Denmark; 2 Emergency Medicine and Emergency Medical Services, Head Office, The Capital Region of Denmark, Kongens Vænge 2, Hillerød 3400, Denmark

**Keywords:** Emergency Medical Services, Training, Basic Life Support, Manikin

## Abstract

**Background:**

Ambulance personnel play an essential role in the ‘Chain of Survival’. The prognosis after out-of-hospital cardiac arrest was dismal on a rural Danish island and in this study we assessed the cardiopulmonary resuscitation performance of ambulance personnel on that island.

**Methods:**

The Basic Life Support (BLS) and Automated External Defibrillator (AED) skills of the ambulance personnel were tested in a simulated cardiac arrest. Points were given according to a scoring sheet. One sample *t* test was used to analyze the deviation from optimal care according to the 2005 guidelines. After each assessment, individual feedback was given.

**Results:**

On 3 consecutive days, we assessed the individual EMS teams responding to OHCA on the island. Overall, 70% of the maximal points were achieved. The hands-off ratio was 40%. Correct compression/ventilation ratio (30:2) was used by 80%. A mean compression depth of 40–50 mm was achieved by 55% and the mean compression depth was 42 mm (SD 7 mm). The mean compression rate was 123 per min (SD 15/min). The mean tidal volume was 746 ml (SD 221 ml). Only the mean tidal volume deviated significantly from the recommended (p = 0.01).

During the rhythm analysis, 65% did not perform any visual or verbal safety check.

**Conclusion:**

The EMS providers achieved 70% of the maximal points. Tidal volumes were larger than recommended when mask ventilation was applied. Chest compression depth was optimally performed by 55% of the staff. Defibrillation safety checks were not performed in 65% of EMS providers.

## Background

Survival after out-of-hospital cardiac arrest (OHCA) is generally below 10%, a figure that has remained essentially unchanged over the past three decades despite efforts to improve survival [1]. The ‘Chain of Survival’ comprises early recognition of the cardiac arrest, early cardiopulmonary resuscitation (CPR), early defibrillation and post-resuscitation care, which are the actions needed for successful resuscitation [2]. The quality of CPR appears to impact the survival [3-5], but unfortunately the CPR quality has been shown to be poor and not consistent with international guidelines for resuscitation, even when performed by health care professionals [6-8].

On the rural Danish island of Bornholm no one survived an OHCA in 2001–2003 [9]. Therefore a multifaceted approach was launched in September 2008 with the purpose to increase the survival after OHCA by improving the quality of each link in the ‘Chain of Survival’. The Emergency Medical Service (EMS) providers are an important part of this by providing CPR and defibrillation, especially in rural areas with long transportation times. Little is known about the resuscitation skills in low-volume EMS as most research performed in the area is conducted in larger cities. The aim of this study was to determine the Basic Life Support (BLS) and Automated External Defibrillator (AED) skills in the EMS providers on the island. Thereby we would be able to intervene where needed and hopefully also improve the quality of care.

## Methods

### Setting

Bornholm is an island of 588 km^2^ with a population of 42.000.The number of OHCA is approximately 50 cases per year.

### EMS system

At the time of the investigation (May 2009) the EMS response to presumed OHCA was an ambulance unit manned with two BLS providers trained in the use of a defibrillator in AED-mode. The unit was dispatched from two different locations simultaneously with a BLS provider operated first responder unit. There was no paramedic-, nurse- or physician-manned ambulances on the island.

There were 22 EMS providers on the island educated as ‘level 2’ providers and 13 educated as ‘level 1’ providers. All are trained in BLS and AED use, but the level 2 providers have received additional training in ECG interpretation and completed the international certified Pre-Hospital Trauma Life Support course [10]. The EMS providers work together in pairs on a 24-hour shift every third day. During each shift one EMS provider is assigned the leader and the other an assistant. The assistant may be a ‘level 1’ EMS provider, but the leader has to be a ‘level 2’ provider.

OHCA was treated in accordance with the European Resuscitation Council (ERC) Guidelines for Resuscitation 2005. The treatment of unwitnessed OHCA was 2 min of CPR prior to rhythm analysis [11].

### BLS/AED assessment

On the 25th, 26th and 27th of May 2009 we assessed the BLS and AED skills of the individual EMS teams responding to OHCA on the island.

The teams were called one by one to the main station during their shift and were told that they had to participate in a simulated cardiac arrest situation. They were to bring all their usual equipment and the scenario was: “An adult citizen has called the dispatch centre and reported that he is unwell and alone in his house. Upon arrival you find the citizen lying on the floor, apparently without signs of life. There is no one else around.”

Ideally the EMS team should recognise cardiac arrest, start and continue CPR for 2 min while attaching the defibrillator (Physio-Control LifePak 15) in AED-mode. A rhythm check should be performed while ensuring nobody touched the victim. In the scenario the rhythm was shockable and a safety check should be performed before pushing the shock button. Then CPR should be performed for another 2 min and the test was stopped just after the second rhythm check.

All ‘level 2’ providers were tested as the leader. The assistant was either a ‘level 1’ or a ‘level 2’ provider, according to the on-duty schedule the specific day. The roles were predefined; the assistant provided chest compressions and maybe ventilations for the first 2 min and after that, only chest compressions.

Skills were assessed on a resuscitation manikin (ResusciAnne HLR-D, Laerdal Medical, Stavanger, Norway) and in accordance with the ERC Guidelines for Resuscitation 2005 [12]. A laptop running Laerdal PC SkillReporting System version 2.0 (Laerdal Medical, Stavanger, Norway) was connected to the manikin and registered the data on hand placement, compression depth, total number of compressions and ventilations, ventilation volume, total hands-off time, delay until first compression or ventilation and time until first shock.

An ERC-certified BLS/AED instructor, ALS provider, and medical doctor obtained ordinal data and registered these on a form during assessment. The following variables were collected: checking for responsiveness by talking and shaking, opening the airway, checking for respiration and pulse, starting BLS, performing safety checks, removing oxygen during shock and compression/ventilation ratio. Before the assessment it was decided that; ideally BLS should be initiated within 15 sec, delay was the time until first compression or ventilation, hand position was incorrect if one compression was in the wrong position, correct compression:ventilation ratio was 30 ± 2:2, and participants who attempted a ratio of 30:2 were registered as such even if ventilations were unsuccessful. There were 2x2 min for compressions and ventilations, thus an optimal 300 compressions and 20 ventilations could be performed within this timeframe. The hands-off time was defined as the time without compressions being performed and the hands-off ratio was the hand-off time divided by the scenario length.

A scoring sheet was developed from the Cardiff test [13] and the scores allocated to each of the 22 categories can be seen in Additional file 1. The total score ranged from 19–70. The percentage of the maximal achievable was calculated by dividing the average score (minus 19) with the maximal score (70–19).

### Statistics

Data are reported as mean with standard deviation (±SD). Descriptive statistics were used to characterize the sample and each of the questions. We decided that the mean tidal volume should not exceed 600 ml, the mean compression depth should be more than 40 mm, the mean total number of ventilations should be below 20 and the mean compression rate should not exceed 120/min. To test if the performance of the EMS providers deviated significantly from these values a one sample *t* test was performed. P-values below 0.05 were considered statistically significant.

### Feedback

After each assessment, individual feedback was given to the EMS providers. Good performance was pointed out and the skills that needed improvement were identified and explained. This was done by the medical doctor who had also performed the assessment.

## Results

We tested 20 (91%) of the 22 level 2 EMS providers on Bornholm as the leader; one was on holiday and one was absent due to illness. Seventeen (85%) were male. Thirteen level 1 providers participated as assistants. The obtained skills can be seen in Additional file 1. The mean total score was 55 (±4) equal to 70% of the maximal achievable score according to the Cardiff test.

The mean delay from the start of the scenario to the first compression or ventilation was 35 (±9) sec and the mean time until the first shock was delivered was 162 (±52) sec.

The mean hands-off time was 122 (±25) sec and the hands-of ratio was 40%. The mean scenario length was 303 (±54) sec.

**Figure 1:**
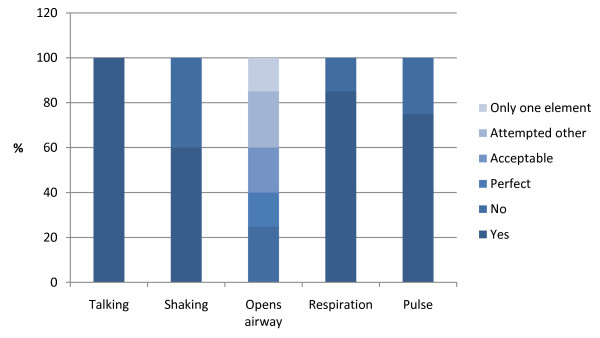
Recognition of cardiac arrest by ambulance personnel (N = 20).

Data on recognition of cardiac arrest can be seen in Figure 1. Of the 75% who checked pulse, all but one (radial artery) used the carotid artery.

### Algorithms of resuscitation

After recognition of cardiac arrest all started BLS and 50% continued for the recommended 2 min. Eighty percent used the correct compression:ventilation ratio (30:2), one was using the ratio from Guidelines 2000 (15:2) and 3 were only doing compressions. The person giving chest compressions was changed every 2 min by 35%. All switched on the AED, but after defibrillation, 15% checked the rhythm, pulse or other signs of life instead of continuing CPR immediately.

### Compressions

**Figure 2:**
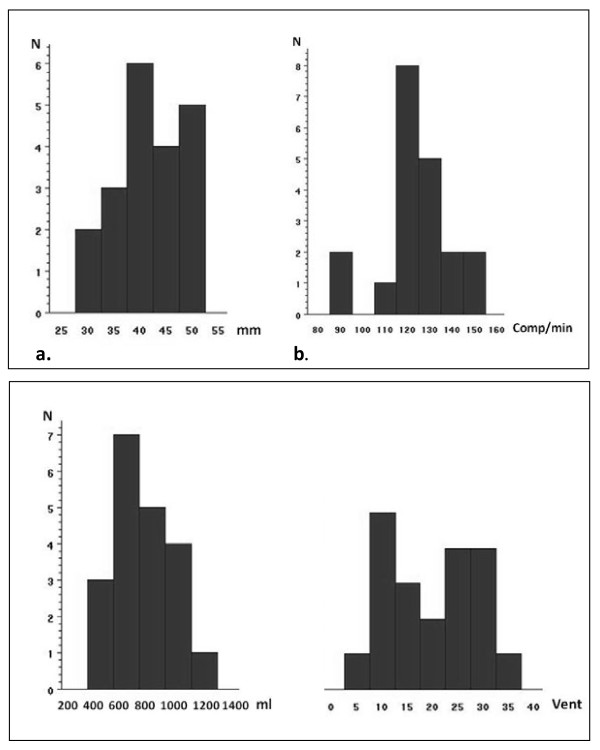
**Compression depth and rate.** Part a: The mean compression depth (mm) performed by the ambulance personnel (N = 20). The recommended compression depth in guidelines 2005 for resuscitation was 40–50 mm and 55% achieved this. Part b: The mean compression rate (compressions/min) performed by the ambulance personnel (N = 20). The optimal compression rate according to guidelines 2005 for resuscitation was 100/min.

The hand-placement was correct for 45% of the EMS providers. The mean compression depth was 42 (±7) mm (Figure 2) and not significantly different from the minimum value of 40 mm (p = 0.26). Likewise, the mean compression rate of 123 (±15)/min (figure 2) was not significantly different from our defined maximum compression rate of 120 (p = 0.37), but 10 of 20 participants had rates higher than the maximally allowed.

The mean number of compressions given in the 2x2 min of BLS was 345 (±95).

### Ventilations

**Figure 3:**
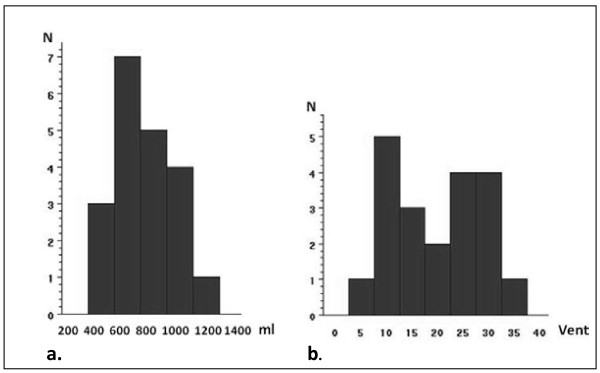
**Tidal volume and number of ventilations.** Part a: The mean tidal volume (ml) performed by the ambulance personnel (N = 20). The recommended tidal volume according to guidelines 2005 for resuscitation was 500–600 ml and 10% achieved this. Part b: The total number of ventilations performed by the ambulance personnel (N = 20). In our scenario the optimal number of ventilations was 20.

The mean tidal volume was 746 (±221) ml (Figure 3) and significantly higher than 600 ml (p = 0.01). Seventeen of 20 participants had ventilation volumes higher than 600 ml. A mean of 20 (±9) ventilations (figure 3) was given in the 2x2 min of BLS, which was not significantly different from 20 (p = 0.8).

### Defibrillation

**Figure 4:**
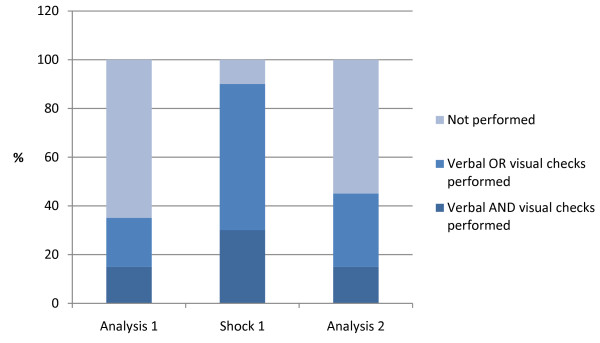
**Safety checks.** Safety checks performed by the ambulance personnel (N = 20) during Automated External Defibrillator analysis and before pushing the ‘shock’ button.

Data on safety checks when operating the AED can be seen in Figure 4. All removed the oxygen mask, but 90% removed it less than 1 meter.

## Discussion

In this assessment of the BLS and AED skills in the cohort of EMS providers in a local low-volume Scandinavian ambulance setting, 70% of the maximal achievable score according to the Cardiff test was reached. There is, however, room for improvements. Compressions were given at a higher rate than recommended in the guidelines but compression depth was adequate in a majority of cases. Ventilation was successful but tidal volume was significantly higher than the maximum recommended value. All managed to deliver a defibrillation shock, but the safety checks in relation to AED managing were not satisfactory in all subjects.

The strengths of our study include that the study population comprised 91% of the entire cohort of EMS providers ‘level 2’ in the community and thus gives a realistic assessment of the available resuscitation skills. The ordinal data was registered by one doctor only, thus avoiding inter observer variability. Given that the study was performed with a manikin and not actual cardiac arrest victims, one could argue that this dummy setting does not extrapolates to real life. On the other hand, this design enabled us to study variables, which has not been studied in real life settings, like the recognition of cardiac arrest and safety checks related to the AED deployment. Also our measurements of variables such as ventilation volume and compression depth might be more consistent. A limitation is that the EMS providers knew that they were to participate in a research project regarding resuscitation skills. Another limitation is that it would have been beneficial to create a specific course where the identified skills needing improvement could have been trained systematically and afterwards re-assessed. Unfortunately, for logistics and economic reasons this was not possible. One could argue that our emphasis on safety checks is rather large, since the EMS providers often are performing resuscitation alone in the vehicle during transport. But in the patients home they are working together and likely with relatives present.

Throughout the ERC guidelines for resuscitation 2005 and 2010 [14,15] there is increased emphasis on minimally interrupted high-quality chest compressions and both human [16] and animal [17,18] studies have shown that even short interruptions in chest compressions are associated with worse outcome.

Previously the quality of real life EMS CPR has been found to be poor with no compressions being given 48% of the time (38% when subtracting time for defibrillation) [6] and 57% of the time, respectively [8]. More recent studies have shown improvements in the hands-off ratios following the Guidelines 2005 for resuscitation, from 23% to 14% [19], and 49% to 34% [20], respectively.

The hands-off ratio in our study was 40%. Some time without chest compressions is unavoidable (e.g. for recognition of cardiac arrest and defibrillation) but the above mentioned clinical studies do not take this into account. Therefore, the hands-off ratio in our study is high, especially since it was a manikin study with no interruptions due to placement of i.v. lines or loading of the patient into the EMS vehicle. In a recent Danish study exactly the loading of the patient was found to be a major contributor to hands-off time [21]. Thus, one of the very important aspects of the guidelines (minimizing the hands-off time) was not performed well by the EMS providers.

Another important parameter discussed in the guidelines is the quality of chest compressions. In our study 55% achieved a compression depth of 40–50 mm, which was the recommended depth in guidelines 2005 [12]. The average compression depth was not significantly too shallow, but still 30% provided compressions that were too shallow. One study has shown that shallow compressions were associated with defibrillation failure [16] and other studies have shown that increasing compression depth was correlated with increasing short-term survival [22,23]. Clinical studies have documented prevalence of too shallow compressions [6,7,24] whereas EMS manikin studies have reported that up to 50% of the compressions were too deep [25,26]. Thus our study is not concurrent with other manikin studies and points out a skill that needs improvement. The mean compression rate was 123 (±15)/min, which was too high but in accordance with other studies [19,23,24]. Only 35% changed the person providing chest compressions every 2 minutes, which is emphasized in the guidelines and only 45% had a correct hand position.

In our study the mean tidal volume was 745 (±221) ml, and significantly exceeding the recommended maximum of 600 ml. Only 10% reached the recommended ventilation volume (500–600 ml) and 75% hyperventilated the manikin. In porcine models hyperventilation has resulted in increased intrathoracic pressure, decreased coronary perfusion pressure and survival rate [27].

A fundamental rule in all first aid is ‘safety first’ and therefore it is surprising that more than half of the EMS providers did not perform any hands-off checks during rhythm analysis and only 30% performed both a visual *and* a verbal hands-off safety check before pushing the shock button. During training, safety checks in relation to the AED should be reinforced.

With regard to recognition of cardiac arrest it is recommended in the guidelines that looking for signs of life should take no more than 10 sec [14]. In our study the delay from start of the scenario to the first compression or ventilation was 35 (±9) sec. Studies with trained laypersons have reported similar excessive times (29–40 sec) for recognition of cardiac arrest [28-30]. When recognizing cardiac arrest 25% did not make any attempt at opening the airway which is higher than the reported 11% in a manikin study with trained emergency healthcare professionals [25]. After recognizing the unwitnessed cardiac arrest, only half performed BLS for 2 min, which was the current guideline. With regard to correct compression/ventilation ratio (30:2) is it surprising that only 80% were able to recall this, 3½ years after the guidelines changed. An explanation could be that the island’s ambulance company (Falck a/s) had a delay of at least 1½ year in implementing the guidelines 2005 [31]. All the issues mentioned in this paragraph are simple cognitive skills and one could speculate if enough attention has been paid to maintenance of EMS providers’ resuscitation skills.

When evaluating the BLS/AED skills in the cohort of EMS providers in a rural low-volume Scandinavian community, 70% of the maximal achievable score according to the Cardiff test was reached. These are the health care professionals who are on duty with the responsibility of taking care of a real cardiac arrest and one could expect a better performance, especially given the inoffensive training scenario. The study took place on a rural island with only approximately 50 cardiac arrests annually, calling for frequent training in BLS/AED. Our findings suggest that this training most likely has been too moderate and in that way our results might extrapolate to the rest of the country as the island’s EMS operator covers approximately 85% of the population in Denmark. In addition, our findings might be generalizable to other similar rural settings in Scandinavia.

Identifying suboptimal performance demands action. The EMS providers should be trained at regular intervals in realistic settings and with qualified instructors. Personnel employed in rural areas might benefit from a rotation system with shifts in more busy areas. This study points out specific difficulties to which training should be targeted. For instance, the recognition of cardiac arrest probably deserves more attention in resuscitation training, including stressing the importance of checking the airways. The manikin was ventilated successfully, but with high tidal volumes. In addition, the tidal volumes were given with very large variability, indicating that this is another skill that should be trained carefully.

## Conclusion

In conclusion, the EMS providers in a rural low-volume Scandinavian setting achieved 70% of the maximal points when their BLS and AED skills were tested in a simulated cardiac arrest. In order to improve the quality of care, future training should be targeted to recognition of cardiac arrest, minimizing the hands-off time, and providing ventilation with adequate tidal volumes. Defibrillation safety checks should be reinforced.

## Abbreviations

AED, Automated External Defibrillator; BLS, Basic Life Support (BLS); CPR, Cardiopulmonary Resuscitation; EMS, Emergency Medical Service; ERC, European Resuscitation Council; OHCA, Out-of-hospital cardiac arrest; SD, Standard deviation.

## Competing interests

The authors declare that they have no competing interests.

## Authors' contributions

All authors made contributions to conception and design and interpretation of data. AMN acquired the data, drafted the manuscript and AMN and LSR performed the statistical analysis. LSR, DLI and FKL have revised the manuscript critically for important intellectual content. All authors read and approved the final manuscript.

## Supplementary Material

Additional file 1 **Assessment of resuscitation skills in ambulance personnel.** Assessment of resuscitation skills in ambulance personnel with points allocated to the different resuscitation skills. Click here for file
