# Supplementary material for: Basic life support and automated external defibrillator skills among ambulance personnel: a manikin study performed in a rural low-volume ambulance setting
Source: Scand J Trauma Resusc Emerg Med. 2012 May 8;20:34. doi: 10.1186/1757-7241-20-34 (PMC3430550; doi:10.1186/1757-7241-20-34)
Supplement: Additional file 1 — Assessment of resuscitation skills in ambulance personnel. Assessment of resuscitation skills in ambulance personnel with points allocated to the different resuscitation skills. [file 1757-7241-20-34-S1.pdf]

Additional file 1. Assessment of resuscitation skills in ambulance personnel.

	**EMS provider** (N=20) N (%)
**Checks responsiveness by talking***	20 (100)
**Checks responsiveness by shaking**	
**3:** Yes	12 (60)
**2:** No	8 (40)
**1:** Potentially dangerous	0
**Opens airway – head tilt, chin lift**	
**5:** Perfect	3 (15)
**4:** Acceptable	4 (20)
**3:** Attempted other	5 (25)
**2:** Only one element	3 (15)
**1:** No	5 (25)
**Checks respiration – see, listen and feel***	17 (85)
**Checks pulse**	
**3:** Checked carotid	14 (70)
**2:** Checked other	1 (5)
**1:** No	5 (25)
**BLS is started**	
**4:** Yes, and continued for 2 min	10 (50)
**3:** Yes, but continued less than 2 min	5 (25)
**2:** No, instead AED is attached without ongoing BLS	5 (25)
**1:** No, BLS not started	0
**Switch on AED***	20 (100)
**Visual and verbal hands-off check during AED analysis (shock 1)**	
**3:** Verbal AND visual checks performed	3 (15)
**2:** Verbal OR visual check performed	4 (20)
**1:** Not performed	13 (65)
**Oxygen mask removed during defibrillation**	
**3:** Yes, ≥ 1 m	2 (10)
**2:** Yes, but < 1 m	18 (90)
**1:** No	0
**Hands off safety check before pushing "shock" button (shock 1)**	
**4:** Verbal AND visual checks, shock button pushed	6 (30)
**3:** Verbal OR visual check, shock button pushed	12 (60)
**2:** No safety checks and shock button pushed	2 (10)
**1:** Shock button not pushed	0
**Post-shock immediately resumption of CPR for 2 min.**	
**4:** Yes, and continued for 2 min	17 (85)
**3:** Yes, but continued less than 2 min	0
**2:** No, shock followed by rhythm check or a check for signs of life or a pulse	3 (15)
**1:** No, BLS not resumed	0
**Visual and verbal hands-off checks during AED analysis (shock 2)**	
**3:** Verbal AND visual checks performed	3 (15)
**2:** Verbal OR visual check performed	6 (30)
**1:** Not performed	11 (55)
**Ventilation/compression ratio**	

**4:** 2:30 (2:28-32)<br>**3:** Other<br>**2:** Compressions only<br>**1:** Ventilations only	16 (80)<br>1 (5)<br>3 (15)<br>0
**The person giving chest compressions are changed every 2 min***	7 (35)
**Hand-placement during compression**<br>**4:** Correct<br>**3:** Other wrong<br>**2:** Too low<br>**1:** Not attempted	 9 (45)<br>10 (50)<br>1 (5)<br>0
**Average compression depth**<br>**6:** 40-50 mm<br>**5:** 51-54 mm<br>**4:** 25-39 mm<br>**3:** ≥55 mm<br>**2:** ≤24 mm<br>**1:** Not attempted	 11 (55)<br>3 (15)<br>6 (30)<br>0<br>0<br>0
**Total compressions counted**<br>**6:** 241-300<br>**5:** ≥301<br>**4:** 181-240<br>**3:** 121-180<br>**2:** 1-120<br>**1:** Not attempted	 2 (10)<br>14 (70)<br>3 (15)<br>1 (5)<br>0<br>0
**Average tidal volume (ml)**<br>**5:** 500-600<br>**4:** ≤499<br>**3:** ≥601<br>**2:** 0<br>**1:** Not attempted	 2 (10)<br>3 (15)<br>15 (75)<br>0<br>0
**Total ventilations counted**<br>**5:** 12-20<br>**4:** 1-11<br>**3:** ≥21<br>**2:** 0<br>**1:** Not attempted	 4 (20)<br>6 (30)<br>10 (50)<br>0<br>0
**Total score**	55 (4)

Additional file 1. Assessment of resuscitation skills in ambulance personnel. Results are shown as number (%). The total score is reported as mean with (SD).

*the points are: **2:** Yes, **1:** No. The reported proportion is the "Yes" category.
